# Functional characterization of two 3-dehydroquinases of AroQ1 and AroQ2 in the shikimate pathway and expression of genes for the type III secretion system in *Ralstonia solanacearum*

**DOI:** 10.3389/fmicb.2023.1186688

**Published:** 2023-04-26

**Authors:** Qingshan Zhang, Bofan Wu, Liangliang Han, Duan Yu, Tao Liang, Yan Wang, Tao Guo

**Affiliations:** ^1^College of Resources and Environment, Southwest University, Chongqing, China; ^2^Chongqing Academy of Agricultural Sciences, Chongqing, China

**Keywords:** *Ralstonia solanacearum*, type three secretion system (T3SS), pathogenesis, global regulation network, biosynthesis of aromatic amino acids

## Abstract

The shikimate pathway is a general route for the biosynthesis of aromatic amino acids (AAAs) in many microorganisms. A 3-dehydroquinase, AroQ, controls the third step of the shikimate pathway that catalyzes the formation of 3-dehydroquinate from 3-dehydroshikimate via a trans-dehydration reaction. *Ralstonia solanacearum* harbors two 3-dehydroquinases, AroQ1 and AroQ2, sharing 52% similarity in amino acids. Here, we demonstrated that two 3-dehydroquinases, AroQ1 and AroQ2, are essential for the shikimate pathway in *R. solanacearum*. The growth of *R. solanacearum* was completely diminished in a nutriment-limited medium with the deletion of both *aroQ1* and *aroQ2*, while substantially impaired *in planta*. The *aroQ1/2* double mutant was able to replicate i*n planta* but grew slowly, which was ~4 orders of magnitude less than the parent strain to proliferate to the maximum cell densities in tomato xylem vessels. Moreover, the *aroQ1/2* double mutant failed to cause disease in tomato and tobacco plants, whereas the deletion of either *aroQ1* or *aroQ2* did not alter the growth of *R. solanacearum* or pathogenicity on host plants. Supplementary shikimic acid (SA), an important intermediate of the shikimate pathway, substantially restored the diminished or impaired growth of *aroQ1/2* double mutant in a limited medium or inside host plants. The necessity of AroQ1 and AroQ2 on the pathogenicity of *solanacearum* toward host plants was partially due to insufficient SA inside host plants. Moreover, the deletion of both *aroQ1* and *aroQ2* significantly impaired the expression of genes for the type III secretion system (T3SS) both *in vitro* and *in planta*. Its involvement in the T3SS was mediated through the well-characterized PrhA signaling cascade and was independent of growth deficiency under nutrient-limited conditions. Taken together, *R. solanacearum* 3-dehydroquinases play important roles in bacterial growth, the expression of the T3SS, and pathogenicity in host plants. These results could extend our insights into the understanding of the biological function of AroQ and the sophisticated regulation of the T3SS in *R. solanacearum*.

## Introduction

*Ralstonia solanacearum* is a soil-borne plant pathogenic bacterium that is a causal agent of bacterial wilt on over 450 plant species belonging to 50 botanical families worldwide and causes severe economic losses in many economically important crops, such as tomato, eggplant, potato, tobacco, and banana worldwide (Mansfield et al., 2012; Jiang et al., 2017). The infection process of *R. solanacearum* toward host plants involves several stages, including soil survival and moving to host rhizosphere soil, root attachment, colonization of root cortex, moving into developing vascular bundles, and spreading systemically into xylem vessel (McGarvey et al., 1999; Caldwell et al., 2017). In the xylem vessel, *R. solanacearum* proliferates vigorously and produces a huge number of exopolysaccharides slime (EPS) that aids in bacterial adhesion and biofilm formation, further facilitating the colonization of plant tissues, and blocks the water-transporting system, leading to severe water-transport obstruction, characteristic wilting symptoms, and plant death (Roberts et al., 1988; Lowe-Power et al., 2018). At the early stage of infection, especially at root attachment, *R. solanacearum* uses a syringe-like type III secretion system (T3SS) to inject virulence factors (so-called type III effectors, T3Es) into host cytosol that usurps and subvert host defense to facilitate bacterial survival and multiplication within host tissues (Genin and Denny, 2012; Coll and Valls, 2013; Deng et al., 2017; Landry et al., 2020). The T3SS in *R. solanacearum* is encoded by 22 genes forming a *hrp* regulon that is greatly conserved among *Ralstonia solanacearum* species complex (RSSC), which is extremely heterogeneous and includes *R. solanacearum* and closely related species of *R. syzygii, R. pickettii*, and banana blood disease bacterium (Fegan and Prior, 2005; Genin and Denny, 2012; Coll and Valls, 2013). Transcription of the T3SS and abundant T3Es genes in RSSC is directly controlled by a key regulator HrpB, an AraC-type of transcriptional regulator, which binds directly to the *hrp*_*II*_ motif in promoters (Cunnac et al., 2004; Mukaihara et al., 2010). Transcription of *hrpB* and T3SS genes is activated by host plants or some mimic plant signals, but it is not initiated in nutrient-rich conditions (Arlat et al., 1992; Zhang et al., 2013). Two close paralogs, HrpG and PrhG, which are the OmpR/PhoB family of response regulators of two-component systems (TCSs), positively regulate the expression of *hrpB* and the T3SS genes in parallel ways (Valls et al., 2006; Plener et al., 2010; Zhang et al., 2013). Different from the expressional characteristic of *hrpB* and the T3SS, HrpG and PrhG are well-expressed under nutrient-rich conditions, which can respond to host signals or mimic signals by phosphorylation and then initiate transcriptional regulation on downstream *hrpB* gene (Yoshimochi et al., 2009a,b; Plener et al., 2010; Zhang et al., 2013). HrpG responds to host signals and plays essential roles in host invasion, colonization, and pathogenicity, while PrhG responds to some metabolic signals and contributes partially to host colonization and pathogenicity (Plener et al., 2010; Zhang et al., 2013; Pedro-Jove et al., 2021). Host plant signals or mimic signals are perceived by an outer membrane protein PrhA and transferred to HrpG via a signaling cascade of PrhA-PrhI/R-PrhJ, while it remains unclear how PrhG responds to its signals (Valls et al., 2006; Pedro-Jove et al., 2021). Intriguingly, a global virulence regulator PhcA, which is a LysR-type transcriptional regulator and is quorum sensing-dependent, regulates the expression of *hrpG* and *prhG* in a contrasting way. PhcA represses *prhIR* expression at high cells density by binding its promoter and it in turn negatively regulates *hrpG* and *hrpB* expressions, while PhcA positively regulates *prhG* expression through some novel pathways (Genin et al., 2005; Yoshimochi et al., 2009a; Zhang et al., 2013, 2015). *R. solanacearum* might switch from using HrpG to PrhG to activate *hrpB* expression in a cell density-dependent manner (Zhang et al., 2013).

We previously demonstrated that two DAHP synthases, AroG1 and AroG2, are essential for the biosynthesis of aromatic amino acids (AAAs), L-phenylalanine (Phe), L-tyrosine (Tyr), and L-tryptophan (Trp) in *R. solanacearum*. Furthermore, they play an important role in the T3SS expression, and their impact on the T3SS is mediated through the well-characterized PrhA-HrpG signaling cascade. The shikimate pathway is a general route for AAAs biosynthesis in many microorganisms that consists of seven steps beginning with the condensation of erythrose 4-phosphate (E4P) and phosphoenolpyruvate (E4P) and ending with the chorismate formation (Sprenger, 2006; Maeda and Dudareva, 2012). DAHP synthases AroG controls the first step in the shikimate pathway that catalyzes the formation of 3-deoxy-D-arabino-heptulosonate-7-phosphate (DAHP) by the condensation of E4P and PEP in many microorganisms (Ogino et al., 1982; Herrmann, 1983). Chorismate is a common precursor leading to the biosynthesis of three AAA and some derivatives, such as vitamin K, ubiquinone, and folic acid (Bentley, 1990; Dosselaere and Vanderleyden, 2001; Gosset et al., 2001). We focused on synthetases in the shikimate pathway, 3-dehydroquinase, i.e., AroQ, which controls the third step of the shikimate pathway and catalyzes the formation of 3-dehydroquinate from 3-dehydroshikimate via a trans-dehydration reaction (Herrmann and Weaver, 1999; Sprenger, 2006; Maeda and Dudareva, 2012). With genome searching, two 3-dehydroquinases, AroQ1 (RSc2785, 156 amino acids) and AroQ2 (RSp1397, 154 amino acids), were annotated in the genome of *R. solanacearum* GMI1000, which share 52% identity on amino acids. We here focused on AroQ1 and AroQ2 to elucidate their functional roles on the shikimate pathway, the T3SS expression, and pathogenicity in *R. solanacearum*.

## Materials and methods

### Bacterial strains and culture conditions

The bacterial strains used in this study are listed in Table 1. *R. solanacearum* strains were derivatives of OE1-1, a phylotype I, race 1, and biovar 4 pathogenic strain on tomato and tobacco plants (Kanda et al., 2003). *E. coli* strains of DH12S and S17-1 were grown at 37°C in the Luria–Bertani medium and used for plasmid construction and conjugation, respectively. *R. solanacearum* strains were grown at 28°C in a nutrient-rich B medium or a minimal medium (Hoagland medium with 2% of sucrose, also used as a *hrp-*inducing medium; Yoshimochi et al., 2009b).

**Table 1:** Bacterial strains used in this study.

**Strain**	**Relative characteristics**	**References**
***Escherichia coli***		
DH12S	Host strain for DNA manipulation	Invitrogen
S17-1	Host strain for plasmid mobilization	Simon et al., 1983
***R. solanacearum***		
OE1-1	Wild-type, race 1, and biovar 3	Kanda et al., 2003
RK5134	OE1-1 and *prhA-lacZYA*	Yoshimochi et al., 2009b
RK5046	OE1-1 and *hrpB-lacZYA*	Yoshimochi et al., 2009b
RK5050	OE1-1 and *popA-lacZYA*	Yoshimochi et al., 2009b
RK5120	OE1-1 and *hrpG-lacZYA*	Yoshimochi et al., 2009b
RK5124	OE1-1 and *prhJ-lacZYA*	Yoshimochi et al., 2009b
RK5130	OE1-1 and *prhIR-lacZYA*	Yoshimochi et al., 2009b
RK5212	OE1-1 and *prhG-lacZYA*	Zhang et al., 2013
RK5619	OE1-1 and *prhN-lacZYA*	Zhang et al., 2015
RQ5920	*popA-lacZYA* and *ΔaroQ1*	This study
RQ5908	*popA-lacZYA* and *ΔaroQ2*	This study
RQ5951	*popA-lacZYA* and *ΔaroQ1/2*	This study
RQ5996	OE1-1 and *ΔaroQ1*	This study
RQ6013	OE1-1 and *ΔaroQ2*	This study
RQ6037	OE1-1 and *ΔaroQ1/2*	This study
RQC1434	OE1-1, *ΔaroQ1/2*, and *hrpB-lacZYA*	This study
RQC1437	OE1-1, *ΔaroQ1/2*, and *hrpG-lacZYA*	This study
RQC1440	OE1-1, *ΔaroQ1/2*, and *prhA-lacZYA*	This study
RQC1443	OE1-1, *ΔaroQ1/2*, and *prhJ-lacZYA*	This study
RQC1446	OE1-1, *ΔaroQ1/2*, and *prhG-lacZYA*	This study
RQC1662	OE1-1, *ΔaroQ1/2*, and *prhIR-lacZYA*	This study
RQC1731	OE1-1 and *ΔaroQ1/2*, and *prhN-lacZYA*	This study
RQC1601	OE1-1 and *ΔaroQ1/2+ aroQ1*	This study

### Mutant generation with an in-frame deletion of target genes

Target genes were in-frame deleted via pK18mobsacB-based homolog recombination as described previously, which is a mark-free and appropriate for multiple deletions of a subset of target genes in *R. solanacearum* (Zhang et al., 2015; Lei et al., 2020). In brief, DNA fragment flanking both ends of the target gene was conjugated with a joint PCR and sub-cloned into pK18mobsacB. The desired plasmid was transferred into *R. solanacearum* strains by conjugation with *E. coli* S17-1, and generated mutants were confirmed by colony PCR with respective primer pairs (Supplementary Table 1).

### Complementation analyses

Genetic complementation was performed with a Tn*7*-based site-specific chromosomal integration system as described previously (Zhang et al., 2015, 2021). In brief, the DNA fragment containing the coding sequence of the target gene and its upstream region of about 500 bp (empirically harboring its native promoter) was PCR amplified and cloned into pUC18-mini-Tn*7*T-Gm (Choi et al., 2005). After validating the sequence, the target DNA fragment was integrated into the chromosome of corresponding mutants at 25-bp of *glmS* downstream as monocopy that was confirmed by colony PCR with primer pair of glmsdown and Tn7R (Zhang et al., 2015, 2021).

### Bacterial growth assay

Bacterial growth was assessed both *in vitro* (rich medium and minimal medium) and *in planta* (tobacco leaves and tomato stems) as described previously (Zhang et al., 2017). Cell densities in tobacco leaves were represented in log_10_ CFU cm^−2^ and that in tomato stems were represented in log_10_ CFU g^−1^. Shikimic acid and L-Phe, L-Tyr, and L-Trp were purchased from Sango Biotech (Shanghai China) and were supplemented into minimal medium and tomato stem to restore the growth of the *aroQ1/2* double mutant. For tomato stems supplementation, tomato cutting seedlings were soaked into one-quarter diluted minimal medium (Hoagland medium) with 200 μM of each L-Phe, L-Tyr, and L-Trp for about 4 days and then inoculated with the petiole-cutting inoculation method. Each assay was performed with three biological replicates including three replicates per trial for the growth assay in medium and four biological replicates including six plants per trial for the *in planta* growth assay. Mean values of all experiments were averaged with SD, and statistical significance was assessed using a *post-hoc* Dunnett's test following ANOVA.

### β-galactosidase assay

Expression levels of *lacZYA*-fused target genes were evaluated with the β-galactosidase assay both *in vitro* and *in planta* as described previously (Zhang et al., 2013). Enzyme activities *in vitro* were expressed in Miller units (Miller, 1992) and that *in planta* was normalized with luminescence divided by cell number (Zhang et al., 2013). Each assay was performed with at least four biological replicates, including four replications per trial. Mean values of all experiments were averaged with SD, and statistical significance was assessed using a *post-hoc* Dunnett's test following ANOVA.

### Virulence assay

Virulence assay was performed on wilt-susceptible tomato (*Solanum lycopersicum* cv. Moneymaker) and tobacco (*Nicotiana tabacum* CV. Bright Yellow) plants, which were grown at 25°C for about 3–4 weeks and subjected to the virulence test (Yao and Allen, 2007; Zhang et al., 2013). Tomato plants were inoculated by the petiole-cutting inoculation method that enables direct invasion into xylem vessels, and tobacco plants were inoculated by the leaf infiltration method that enables direct invasion into intercellular spaces of leaves. For growth restoration with shikimic acid, tomato cutting seedlings were soaked into one-quarter diluted minimal medium with 300 μM of shikimic acid for about 4 days and inoculated with the petiole-cutting inoculation method. Each assay was performed with at least four biological replicates including 12 plants per trial. Wilt symptoms of plants were inspected as a 1–4 disease index, and the mean values of all experiments were averaged. The virulence assay was also presented with survival curves, by which test plants were inspected daily with two levels of no symptoms (disease index below 3) and complete wilting (disease index equal to or higher than 3) as previously described (Poueymiro and Genin, 2009). The statistical significance was assessed using a *post-hoc* Dunnett's test following ANOVA.

## Results

### AroQ1 and AroQ2 control coordinately the biosynthesis of aromatic amino acids via the shikimate pathway in *R. solanacearum*

**Figure 1:**
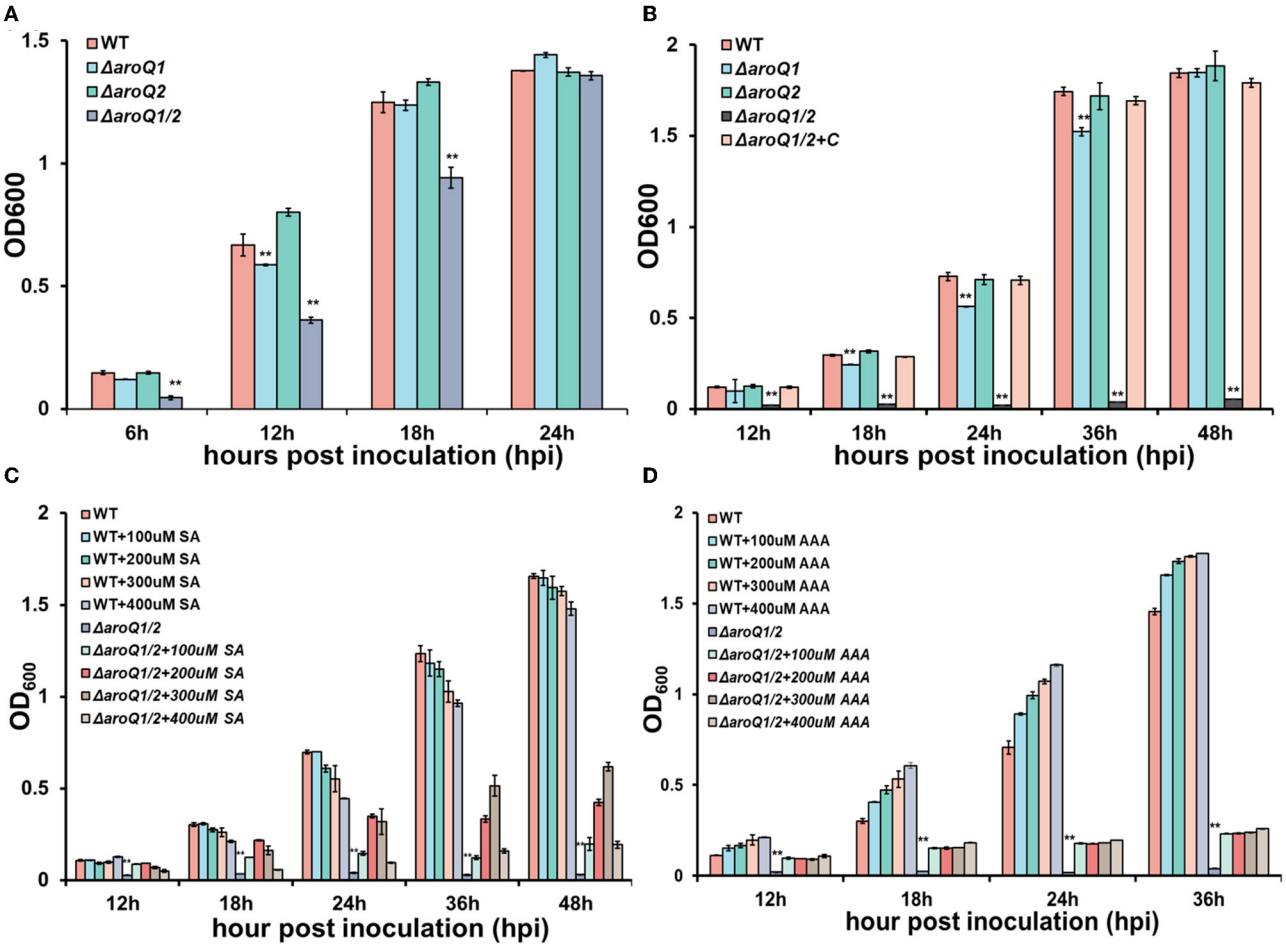
Involvement of AroQ in the growth of *R. solanacearum* OE1-1 in **(A)** nutrient-rich medium, **(B)** nutrient-limited medium (Hoagland medium with 2% of sucrose, minimal medium), **(C)** minimal medium with supplementary shikimic acid (SA) and **(D)** minimal medium with supplementary AAA of L-Phe, L-Tyr, and L-Trp. WT, RK5050 (OE1-1, *popA-lacZYA*); Δ*aroQ1*, RQ5920 (RK5050, Δ*aroQ1*); Δ*aroQ2*, RQ5908 (RK5050, Δ*aroQ2*); Δ*aroQ1/2*, RQ5951 (RK5050, Δ*aroQ1/2*, deletion of both *aroQ1* and *aroQ2*); Δ*aroQ1/2*+C, and RQC1634 (RQ5951 with complementary *aroQ1*). For the growth assay in medium, cell suspension (washed two times with distilled water) was adjusted to an OD_600_ of 1.0 and inoculated into fresh media with a proportion of 1%, and OD_600_ was measured periodically. For supplementation with SA acid and AAA, SA, and AAA (L-Phe, L-Tyr, and L-Trp) were added into the minimal medium at moderate concentrations. Each assay was repeated with three biological replicates including three replicates per trial, and mean values of all experiments were averaged with standard deviation (error bars). Statistical significance between the double mutant (RQ5951) and RK5050 was assessed using a *post-hoc* Dunnett's test following the analysis of variance. Significance level ** indicates *P* < 0.01.

AroQ controls the third step of the shikimate pathway that catalyzes the formation of 3-dehydroquinate from 3-dehydroshikimate via a trans-dehydration reaction (Herrmann and Weaver, 1999; Sprenger, 2006; Maeda and Dudareva, 2012). We first generated *aroQ* mutants with the deletion of either *aroQ1* or *aroQ2* or both genes and assessed their growth profiles in a broth medium (nutrient-rich medium) and a minimal medium (Hoagland medium with 2% of sucrose). The growth of the *aroQ1* mutant (RQ5920) was slightly delayed in both nutrient-rich and minimal media, with cell densities (OD_600_) slightly lower than those of the parent strain (RK5050) at the logarithmic phase, whereas the *aroQ2* mutant (RQ5908) grew normally as parent strain RK5050 in both media (Figures 1A, B). RK5050 is a *popA-lacZYA* reporter strain from *R. solanacearum* OE1-1, which exhibits identical growth profiles and infection process as the wild-type strain OE1-1 toward different host plants (Yoshimochi et al., 2009b). The double mutant (RQ5951, with the deletion of both *aroQ1* and *aroQ2*) grew slowly in the rich medium but failed to grow in the minimal medium (Figures 1A, B). The *aroQ2* mutant grew normally as parent strain RK5050, indicating that existing 3-dehydroshikimate AroQ1 could fulfill the proliferation of *R. solanacearum* under nutrient-limited conditions. Single *aroQ1* was thus selected for genetic complementation. Complementary *aroQ1* fully restored the diminished growth of the double mutant (RQ5951) to that of RK5050 in the minimal medium (Figure 1B).

Shikimic acid (SA) is an important intermediate in the shikimate pathway. We assessed whether AroQ1 and AroQ2 control SA biosynthesis. Supplementary SA significantly restored the diminished growth of double mutant, OD_600_ of which was restored to about 0.6 at 36 h post inoculation (hpi) in the minimal medium with supplementary SA at a final concentration of 300 μM (Figure 1C). The shikimate pathway is a general route for AAA biosynthesis in many microorganisms. We further assessed whether AroQ1 and AroQ2 control AAA biosynthesis via the shikimate pathway. Supplementary AAA, including L-Phe, L-Tyr, and L-Trp at 200 μM each, greatly restored the diminished growth of the double mutant in the minimal medium (Figure 1D), indicating that *R. solanacearum* harbors duplicate 3-dehydroquinases of AroQ1 and AroQ2 to control the shikimate pathway and in turn the AAA biosynthesis.

### Both AroQ1 and AroQ2 are important for the proliferation of *R. solanacearum* inside host plants

**Figure 2:**
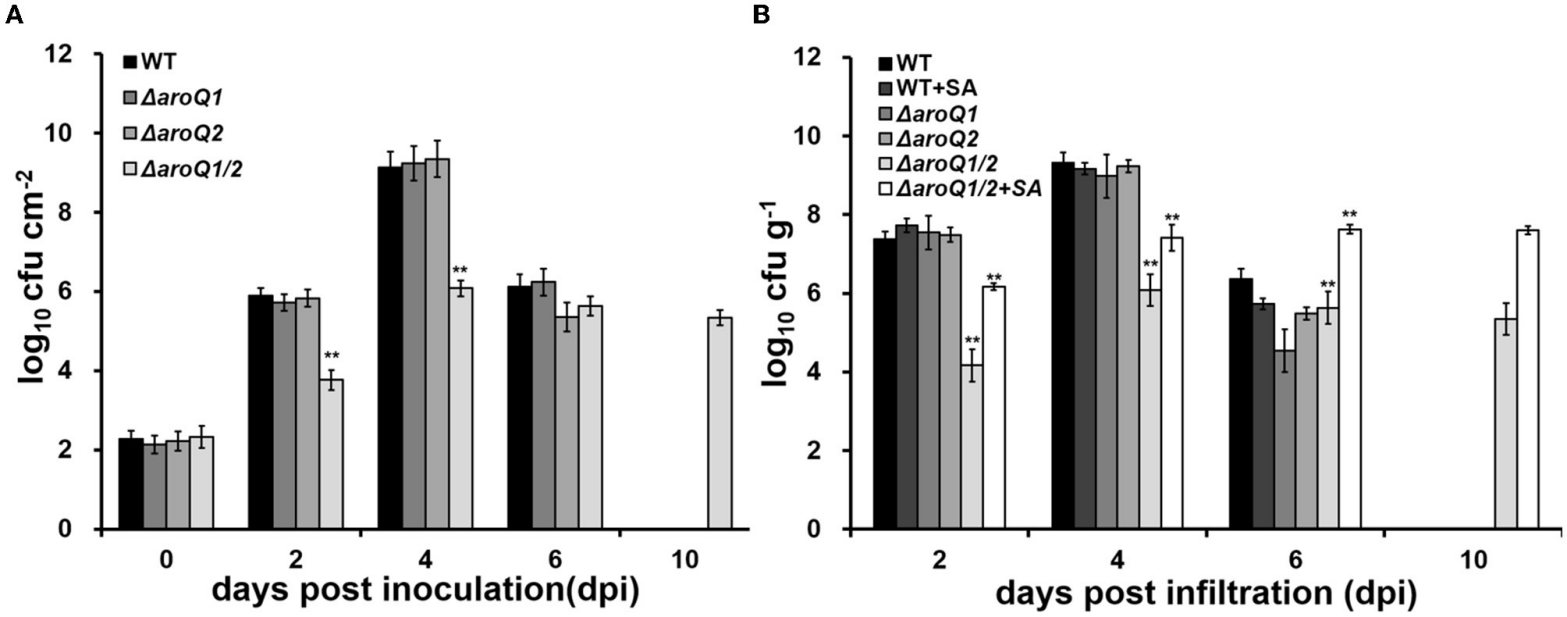
Involvement of AroQ on the growth of *R. solanacearum* OE1-1 in **(A)** tobacco leaves (intercellular spaces) and **(B)** tomato stems (xylem vessels). WT, RK5050 (OE1-1, *popA-lacZYA*); Δ*aroQ1*, RQ5920 (RK5050, Δ*aroQ1*); Δ*aroQ2*, RQ5908 (RK5050, Δ*aroQ2*); Δ*aroQ1/2*, and RQ5951 (RK5050, Δ*aroQ1/2*); +SA, with supplementary shikimic acid (SA). For growth in tobacco leaves, which was presented in log CFU cm^−2^, cell suspension of 0.1 OD_600_ was infiltrated into tobacco leaves, and leaf disks (0.38 cm^2^) were punched for the quantification of cell number by the dilution plating. For the growth assay in tomato stems, which was presented in log CFU g^−1^, tomato plants were inoculated with the petiole-cutting inoculation method, and stem species were cut and weighted for the quantification of cell number by dilution plating. For SA supplementation, shikimic acid was added into a one-quarter diluted minimal medium at 300 Mm, tomato cutting seedlings were soaked for 4 days, and then were inoculated with the petiole-cutting inoculation method. Each assay was repeated with three biological replicates including three replicates per trial for the *in vitro* growth assay, and more than four biological replicates with six plants per trial for the *in planta* growth assay. Mean values from all experiments were averaged and presented with SD, and statistical significance between the double mutant and RK5050 was assessed using a *post-hoc* Dunnett's test following ANOVA. Significance level, ** indicates *P* < 0.01.

Colonization inside host plants, especially extensive proliferation in xylem vessels, is essential for the pathogenicity of *R. solanacearum* (Genin, 2010). Nutrient inside plants is believed to be relatively limited compared with that in rich medium, but relatively more abundant than that in the minimal sucrose medium (Zuluaga et al., 2013; Lowe-Power et al., 2018). We further assessed the proliferation of the *aroQ1* mutant, *aroQ2* mutant, and the double mutant in intercellular spaces of tobacco leaves and tomato xylem vessels. For the growth assay in tobacco leaves, a bacterial suspension at a cell density of 10^4^ CFU ml^−1^ was infiltrated into intercellular spaces of tobacco leaves with a needle-less syringe, and leaf disks were punched for the quantification of cell densities with the dilution plating technique (Zhang et al., 2015). The *aroQ1* mutant and *aroQ2* mutant exhibited similar growth as a parent strain (RK5050) in tobacco leaves, which started at ~10^2^ CFU cm^−2^ on the 1st day (about 6 h after infiltration), drastically reached a maximum of ~10^9^-10^10^ CFU cm^−2^ at 4 days post infiltration (dpi), and then decreased quickly to ~10^6^ CFU cm^−2^ at 6 dpi (Figure 2A). At this point, infiltrated tobacco leaves became withered and dried. The proliferation of double mutant (RQ5951) in tobacco leaves was substantially impaired, which reached the maximum of ~10^6^ CFU cm^−2^ at 4 dpi, but remained at this density till 10 dpi (Figure 2A). At this time point, RQ5951-infiltrated tobacco leaves were healthy, and no wilting symptoms were observed on these leaves.

For the growth assay in tomato stems, tomato plants were inoculated by the petiole-cutting inoculation method, by which 2 μl of a bacterial suspension at 0.1 OD_600_ (10^8^ CFU ml^−1^) was dropped onto the fresh-cut surface of petioles, and stem pieces were excised for the quantification of cells densities using the dilution plating technique (Zhang et al., 2015). The deletion of either *aroQ1* or *aroQ2* did not alter the proliferation of *R. solanacearum* in tomato stems, which proliferated drastically to the maximum of ~10^9^-10^10^ CFU g^−1^ at about 4 dpi and decreased quickly to ~10^5^-10^6^ CFU g^−1^ at 6 dpi (Figure 2B). At this point, RK5050-inoculated plants became collapsed and died. The proliferation of the double mutant (RQ5951) was substantially impaired, which reached to a maximum of about 10^6^ CFU g^−1^ at 4 dpi, but remained at this density till 10 dpi (Figure 2B). At this point, no wilting symptoms were observed on test tomato plants inoculated with the double mutant. Supplementary SA significantly restored the diminished growth of *aroQ1/2* double mutant in the minimal medium. We assessed whether supplementary SA could restore the impaired growth of the double mutant in tomato stems. Tomato cutting seedlings were soaked in one-quarter diluted Hoagland minimal medium with SA (300 μM) for 3 days, which enables tomato plants to accumulate more SA inside stems, and inoculated using the petiole-cutting inoculation method. Supplementary SA did not alter the proliferation of the parent strain (RK5050) in stems of tomato cutting seedlings, but greatly enhanced the growth of double mutant, which was about two orders of magnitude higher than that of the control without SA supplementation, but was about one to two orders of magnitude less than that of RK5050 in tomato stems at 2–4 dpi (Figure 2B). All of these results indicated that both AroQ1 and AroQ2 are important for the proliferation of *R. solanacearum* inside host plants, and the impaired proliferation of the *aroQ1/Q2* double mutant is partially due to insufficient SA inside host plants.

### AroQ1 and AroQ2 are required for the T3SS expression both *in vitro* and *in planta*

**Figure 3:**
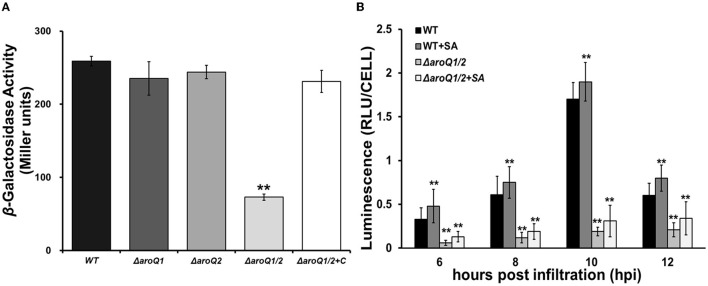
Involvement of AroQ on T3SS expression (*popA-lacZY*A, a representative T3SS gene). **(A)** Expression of *popA-lacZYA* in SA-supplemented *hrp*-inducing minimal medium; **(B)** expression of *popA-lacZYA* in tobacco leaves. WT, RK5050 (OE1-1, *popA-lacZYA*); Δ*aroQ1*, RQ5920 (RK5050, Δ*aroQ1*); Δ*aroQ2*, RQ5908 (RK5050, Δ*aroQ2*); Δ*aroQ1/2*, RQ5951 (RK5050, Δ*aroQ1/2*); Δ*aroQ1/2*+C, and RQC1634 (RQ5951 with complementary *aroQ1*). +SA, supplementary shikimic acid (SA) in tobacco leaves. For the *in vitro* enzyme assay, strains were grown in the *hrp*-inducing minimal medium with supplementary SA at 300 μM to an OD_600_ of 0.1 and subjected to enzymatic measurement, enzymatic activities of which were presented in Miller units. Each assay was repeated with three biological replicates, including three replicates per trial. For the *in planta* enzyme assay, tobacco leaves were infiltrated with a bacterial suspension of 0.1 OD_600_ or with supplementary SA at 300 μM. Leaf disks were punched for enzymatic measurement with the Galacto-Light Plus kit, the enzymatic activities of which were presented with luminescence normalized by cell number (RLU cell−1). The *in planta* enzyme assay was carried out to ~12 h post-infiltration (hpi). At this point, infiltrated regions of tobacco leaves with bacterial cells became clear scald. Luminescence was determined using the GloMax20 Luminometer (Promega), and cell numbers in punched leaf disks were quantified by dilution plating. Each assay was carried out with more than four biological replicates including six replications per trial. Mean values of all experiments were averaged with SD, and statistical significance between mutants and RK55050 was assessed using a *post-hoc* Dunnett's test following ANOVA. Significance level, ** indicates *P* < 0.01.

We previously demonstrated that *R. solanacearum* DAHP synthase AroG controls the shikimate pathway and affects T3SS expression via the PrhA signaling cascade (Zhang W. et al., 2019). We here evaluated whether AroQ1 and AroQ2 affected T3SS expression. The expression of the T3SS genes was monitored with a *popA-lacZYA* fusion, which is located on the left side of the *hrp* regulon and exhibits similar expression profiles as the T3SS genes (Yoshimochi et al., 2009a). Expression of the T3SS was repressed in the rich medium, but induced in the *hrp*-inducing minimal medium or getting in contact with host signals (Valls et al., 2006; Yoshimochi et al., 2009a). The double mutant failed to grow in the *hrp*-inducing minimal medium, and SA (300 μM) was added into the *hrp*-inducing minimal medium to support its growth. Supplementary SA did not alter the *popA* expression in the parent strain RK5050. The *popA* expression in the double mutant (RQ5951) was substantially decreased even in SA-supplemented *hrp*-inducing minimal medium, which was approximately one-third of that in the parent strain RK5050 (Figure 3A). Complementary *aroQ1* fully restored impaired *popA* expression of the double mutant compared to that of RK5050 (Figure 3A). On the contrary, *aroQ1* mutant and *aroQ2* mutant exhibited an identical *popA* expression to RK5050 (Figure 3A).

The T3SS expression can be enhanced to much higher levels in host plants by coming in contact with host signals (Valls et al., 2006), and we further assessed whether AroQ1 and AroQ2 are required for the *in planta* T3SS expression. The *in planta* enzyme assay was carried out in tobacco leaves, and the enzymatic activities of which were presented with RLU cell ^−1^ (luminescence normalized by cell number). A bacterial suspension at 0.1 OD_600_ (10^8^ CFU ml^−1^) was infiltrated into tobacco leaves, and bacterial cells in leaf disks were recovered and subjected to the enzyme assay 6–12 h post-inoculation (hpi). Consistent with the above results from the *in vitro* enzyme assay, the *popA* expression in tobacco leaves was not altered with the deletion of either *aroQ1* or *aroQ2* but diminished in the *aroQ1/2* double mutant, which was < 10% compared to that in RK5050 at 6–12 hpi (Figure 3B). In consideration of the fact that *aroQ1/2* double mutant grew faintly inside host plants and supplementary SA greatly restored its impaired growth in host plants. We assessed whether supplementary SA could restore impaired *popA* expression of the double mutant in tobacco leaves. Bacterial cells were suspended in SA solution (300 μM) at 0.1 OD_600_ and were infiltrated into tobacco leaves for the *in planta* enzyme assay. Supplementary SA slightly enhanced *popA* expression of the parent strain (RK5050) in tobacco leaves, but no restoration in the double mutant at 6–12 hpi (Figure 3B). All of these results indicated that AroQ1 and AroQ2 are required for the T3SS expression both *in vitro* and *in planta*, and their involvement on the T3SS is independent of growth deficiency under nutrient-limited conditions.

### Involvement of AroQ1 and AroQ2 on the T3SS expression was mediated through the PrhA signaling cascade to HrpG and HrpB

The T3SS expression in *R. solanacearum* is directly controlled by a key regulator HrpB, the expression of which is repressed in the rich medium, but activated in the *hrp*-inducing medium (Valls et al., 2006). We deleted *aroQ1* and *aroQ2* from a *hrpB-lacZYA* reporter strain RK5046 to assess the involvement of AroQ on the *hrpB* expression. Consistent with that on *popA* expression, the deletion of either *aroQ1* or *aroQ2* did not alter the *hrpB* expression in the SA-supplemented *hrp*-inducing minimal medium, while the deletion of both *aroQ1* and *aroQ2* substantially decreased the *hrpB* expression (Figure 3A). Complementary *aroQ1* fully restored impaired *hrpB* expression of the double mutant compared to that of the parent strain RK5046, confirming that the impact of AroQ1 and AroQ2 on the T3SS is mediated through the key regulator HrpB.

**Figure 4:**
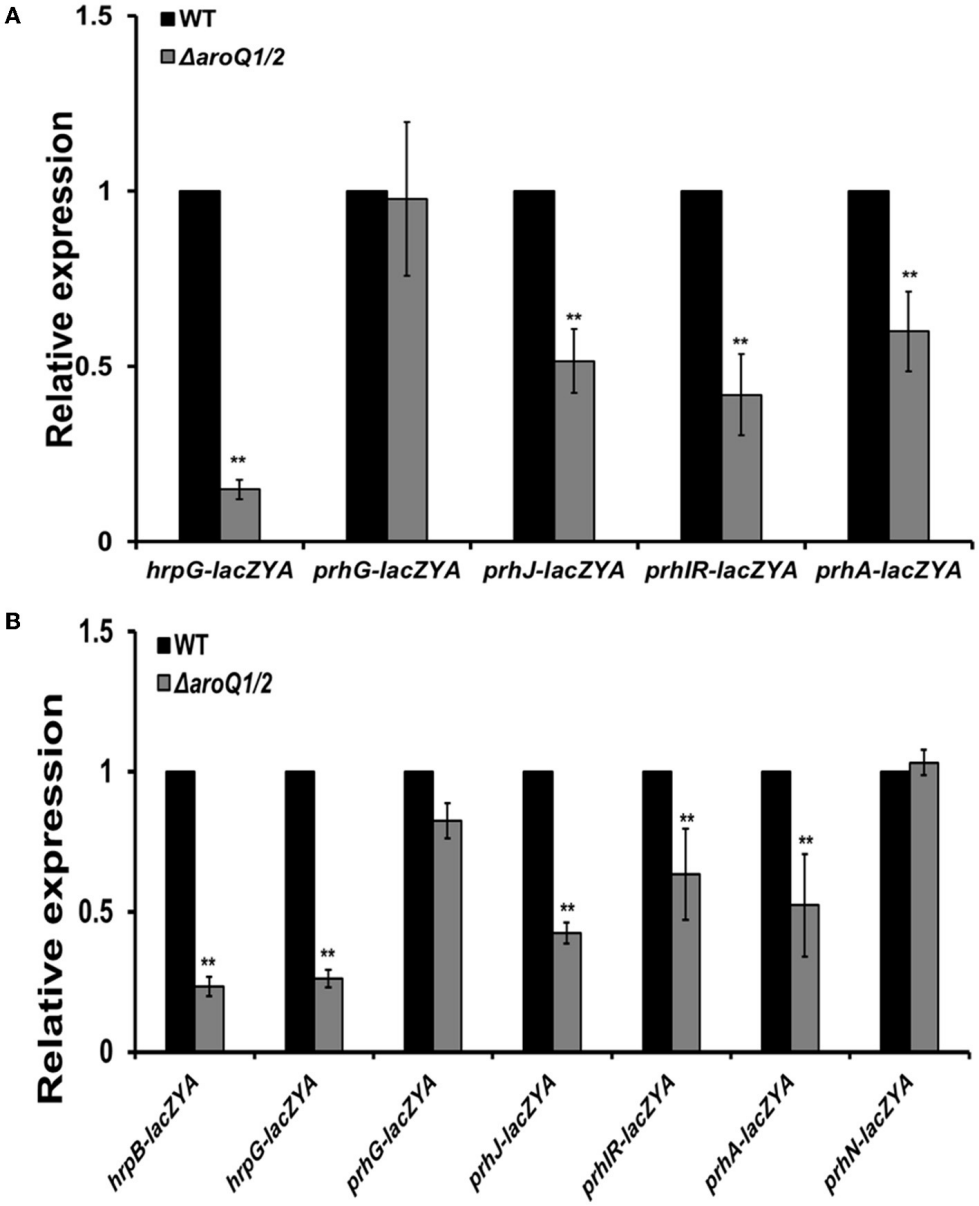
Involvement of AroQ in the expression of T3SS-regulated genes in **(A)** nutrient-rich broth medium and **(B)** in the *hrp*-inducing minimal medium with supplementary SA at 300 μM. WT refers to reporter strains of RK5046 (*hrpB-lacZAY*), RK5120 (*hrpG-lacZAY*), RK5212 (*prhG-lacZAY*), RK5619 (*prhN-lacZAY*), RQ6144 (*prhIR-laczYA*), RQ6074 (*prhJ-lacZYA*), and RK5134 (*prhA-lacZAY*). Δ*aroQ/12* refers to the deletion of both *aroQ1* and *aroQ2* from each reporter strain. The *hrpB* expression was presented only in **(B)** *hrp*-inducing minimal medium since the expression of *hrpB* and the T3SS genes is not activated in a nutrient-rich broth medium. Enzymatic activities were assessed with the *in vitro* enzyme assay and presented in Miller units. Each assay was repeated with three biological replicates including three replicates per trial. Mean values of all experiments were averaged with SD and presented with relative expression compared with their parent reporter strains. Statistical significance between parent strains and the double mutant was assessed using a *post-hoc* Dunnett's test following ANOVA. Significance level, ** indicates *P* < 0.01.

The *hrpB* expression is positively regulated by two close paralogs of HrpG and PrhG in parallel ways (Plener et al., 2010; Zhang et al., 2013). The deletion of *aroQ1* and *aroQ2* substantially impaired *hrpG* expression in the SA-supplemented *hrp*-inducing minimal medium and the rich medium, but no alteration on *prhG* expression (Figures 4A, B). The *hrpG* expression is regulated by a signaling cascade of PrhA-PrhIR-PrhJ. We further generated double mutants from reporter strains of RK5134 (*prhA-lacZYA*), RK5130 (*prhIR-lacZYA*), and RK5124 (*prhJ-lacZYA*) to assess its impact on these T3SS regulating regulators. Expression of these genes was substantially decreased in *aroQ1*/*2* double mutants in the rich medium and the SA-supplemented *hrp*-inducing minimal medium (Figures 4A, B). The *prhG* expression was positively regulated by PrhN and PhcA, respectively, while no alteration in the expression of these genes with the deletion of both *aroQ1* and *aroQ2* (Figures 4A, B). All of these results confirmed that the involvement of AroQ1 and AroQ2 on the T3SS expression is mediated through the PrhA signaling cascade to HrpG and HrpB.

### AroQ1 and AroQ2 were essential for the pathogenicity of *R. solanacearum*

**Figure 5:**
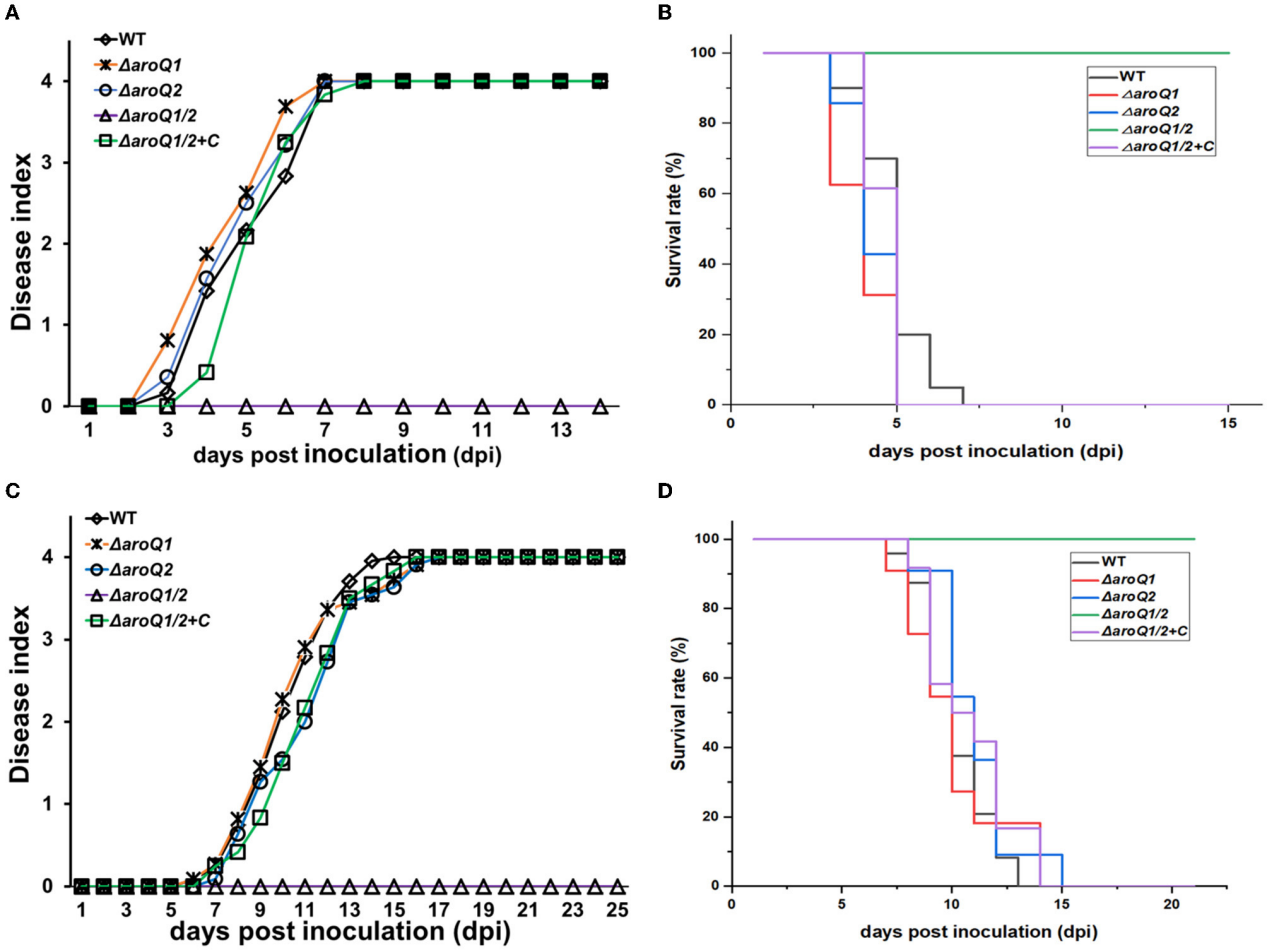
Virulence of *aroQ* mutants on **(A, B)** tomato plants with the petiole-cutting inoculation method **(C, D)**, tobacco plants with the leaf infiltration method. WT, RK5050 (OE1-1, *popA-lacZYA*); Δ*aroQ1*, RQ5920 (RK5050, Δ*aroQ1*); Δ*aroQ2*, RQ5908 (RK5050, Δ*aroQ2*); Δ*aroQ1/2*, RQ5951 (RK5050, Δ*aroQ1/2*); Δ*aroQ1/2*+C, and RQC1634 (RQ5951 with complementary *aroQ1*). **(A, C)** Refer to virulence assay with the disease index. **(B, D)** Refer to surviving curves corresponding to the virulence test with the disease index of **(A, B)**, respectively. For the petiole-cutting inoculation, 2 μl of a bacterial suspension at 10^8^ CFU ml^−1^ was dropped onto the freshly cut surface of tomato petioles. For the leaf infiltration, about 50 μl of the bacterial suspension of 10^8^ CFU ml^−1^ was infiltrated into the tobacco leaves with a blunt-end syringe. For the disease index, wilt symptoms on test plants were scored on a disease index scale from 0 to 4 (0, no wilting; 1, 1–25% wilting; 2, 26–50% wilting; 3, 51–75% wilting; 4, 76–100% wilted and dead). Surviving curves were recorded with two levels of no wilting symptoms (disease index below 3) and completely wilted (disease index equal to or higher than 3) as previously described. Mean values of all experiments were averaged with SD, while it was not presented in figures due to the consideration of esthetic appearance. Statistical significance was assessed using a *post-hoc* Dunnett's test following ANOVA.

The T3SS and *in planta* proliferation are essential for the pathogenicity of *R. solanacearum* to wilt host plants (Genin, 2010). We assessed whether AroQ1 and AroG2 contributed to the pathogenicity of *R. solanacearum* toward different host plants. The virulence assay was performed on host tomato and tobacco plants with the petiole-cutting inoculation method and leaf infiltration methods, respectively, which enable direct invasion into host plants. The *aroQ1* and *aroQ2* mutants exhibited similar virulence as the wild-type strain RK5050 on two host plants, while the *aroQ1/2* double mutant failed to cause any wilting symptoms on two plants till 24 dpi (Figures 5A, B). Complementary *aroQ1* fully restored the diminished pathogenicity of the *aroQ1/2* double mutant compared to that of the wild-type strain (Figures 5C, D), confirming that AroQ1 and AroQ2 are essential for *R. solanacearum* to wilt host plants.

**Figure 6:**
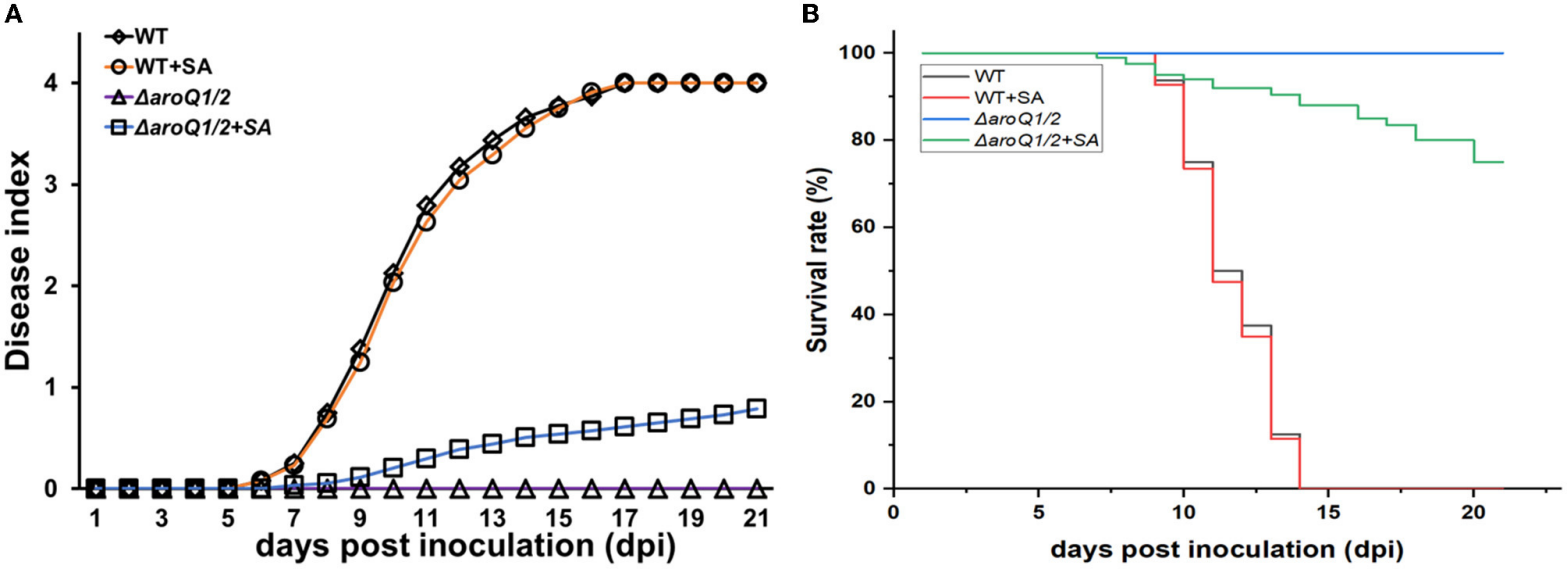
Restoration of SA on the virulence of the *aroQ1/Q2* double mutant on tomato cutting seedlings with petiole-cutting inoculation method. **(A)** Virulence assay with the disease index and **(B)** surviving curves. WT, RK5050 (OE1-1, *popA-lacZYA*); Δ*aroQ1/2*, RQ5951 (RK5050, Δ*aroQ1/2*); +SA, SA supplementation. For SA restoration, tomato cutting seedlings were soaked in one-quarter diluted minimal medium with SA at 300 μM for 4 days and subsequently inoculated with the petiole-cutting inoculation method. The disease index was scored on scales from 0 to 4, and surviving curves were performed with two levels of no wilting symptoms (disease index below 3) and completely wilted (disease index equal to or higher than 3). Mean values of all experiments were averaged with SD, while it was not presented in figures due to the consideration of esthetic appearance. Statistical significance was assessed using a *post-hoc* Dunnett's test following ANOVA.

Supplementary SA substantially restored the proliferation of the *aroQ1/2* double mutant inside tomato stems (Figure 2B). We thus assessed whether the diminished pathogenicity of the *aroQ1/2* double mutant was due to insufficient SA in tomato plants. Tomato cuttings were soaked in one-quarter diluted Hoagland medium with SA (300 μM) for 4 days and subjected to inoculation by the petiole-cutting inoculation method. Supplementary SA did not alter the infection process of the parent strain RK5050 on tomato cuttings (Figures 6A, B). RK5050 wilted tomato cuttings by about 7 dpi and eventually killed all test tomato cuttings by about 14 dpi even without SA supplementation, which was identical to that of RK5050 on tomato cuttings with SA supplementation (Figures 6A, B). Without SA supplementation, the *aroQ1/2* double mutant failed to cause any wilting symptoms on tomato cuttings till 21 dpi, while the double mutant began to wilt about 20% of SA-supplemented tomato cuttings at 21 dpi (Figures 6A, B), indicating that the contribution of AroQ1 and AroQ2 to pathogenicity was partially due to insufficient SA inside host plants.

## Discussion

In the present study, we provided genetic evidence to demonstrate that two putative 3-dehydroquinases, AroQ1 and AroQ2, are cooperatively essential for AAA biosynthesis via the shikimate pathway in *R. solanacearum*. Moreover, they display additional roles in promoting the T3SS expression apart from AAA biosynthesis, and their impact on the T3SS was mediated through the well-characterized PrhA signaling cascade. The shikimate pathway is a common route for AAA biosynthesis in many microorganisms, comprising seven steps, which begins with DAHP formation and ends with chorismate formation (Sprenger, 2006; Maeda and Dudareva, 2012). DAHP synthase AroG controls the first step in the shikimate pathway, and 3-dehydroquinases control the third step in *R. solanacearum*. We previously reported that DAHP synthase AroG1 controls the shikimate pathway and was required for the T3SS expression in *R. solanacearum*. The impact of AroG on the T3SS expression was mediated through the PrhA signaling cascade (Zhang W. et al., 2019). In the shikimate pathway, AroG controls the first step and AroQ controls the third step, respectively, while both of them are required for the T3SS expression via the same PrhA signaling cascade, indicating that enzymes involved in the shikimate pathway might display dual roles in the tryptophan biosynthesis and promoting the T3SS expression in *R. solanacearum*. With genome searching on *R. solanacearum* GMI1000 (a well-studied strain), a subset of enzymes was annotated to be involved in the shikimate pathway, i.e., putative 3-dehydroquinate synthase AroB (RSc2969) controls the second reaction for the formation of 3-dehydroquinate, two shikimate dehydrogenases, AroE1 (RSc2777) and AroE2 (RSp1398), control the forth reaction for the formation of shikimic acid (SA). SA is an important intermediate in the shikimate pathway. Furthermore, shikimate kinase AroK (RSc2970), 1-carboxyvinyltransferase AroA (RSc0907), and chorismate synthase AroC (RSc1566) control the rest three reactions to form chorismic acid, the final product in the shikimate pathway. Further experiments will be performed to confirm whether other enzymes involved in the shikimate pathway display dual roles in the T3SS expression in *R. solanacearum*. Chorismic acid is an unstable branch point intermediate that serves as a precursor leading to all three aromatic amino acids, i.e., anthranilate synthase TrpEG controls the transformation of chorismic acid into anthranilic acid, and finally to tryptophan biosynthesis. We currently demonstrated that TrpE displays dual roles in tryptophan biosynthesis, promoting the T3SS expression in *R. solanacearum*. Different from the shikimate pathway, the impact of TrpE on the T3SS was mediated through parallel pathways of PrhJ-HrpG and PrhG to HrpB (under submission). Moreover, tryptophan plays a role as a novel inducer of the T3SS expression (under submission), while SA and chorismic acid did not alter the T3SS expression (Zhang W. et al., 2019). The *aroQ1/2* double mutant was auxotrophic that failed to grow in nutrient-limited sucrose medium, and supplementary SA could support growth in sucrose medium, while the T3SS expression was substantially impaired with the deletion of both *aroQ1* and *aroQ2* even under SA-supplemented condition, indicating that the impact of AroQ on the T3SS was independent of growth deficiency under nutrient-limited conditions. This phenomenon was also found in some auxotrophic mutants, such as *cysB* mutant (cysteine auxotroph), *proA* mutant (proline auxotroph), and *trpE* mutant (tryptophan auxotroph), which exhibited substantially impaired T3SS expression, but was independent of growth deficiency under nutrient-limited conditions (Chen et al., 2022; Guan et al., 2022). All these suggested that intermediates involved in the shikimate pathway and downstream pathway for AAA biosynthesis might display different functional roles on the T3SS expression. Enzymes involved in the shikimate pathway and the downstream pathway for AAA biosynthesis are required for the T3SS expression, while the impact of these factors on the T3SS expression is mediated through different pathways. Moreover, a PadR-like regulator PrhP displays important roles in the detoxification of ferulic acid and salicylic acid to protect them from the toxicity of phenolic acids, while PrhP positively regulates the T3SS expression mediated through novel pathways to HrpB, which is independent of HrpG and PrhG in *R. solanacearum* (Zhang Y. et al., 2019). All these results confirmed that *R. solanacearum* integrates a complex network to globally regulate the T3SS at different conditions (Genin, 2010; Hikichi et al., 2017). Further experiments are needed to elucidate the global regulation of the T3SS in *R. solanacearum*.

All taken together, these results demonstrated that *R. solanacearum* AroQ displays additional roles in promoting the T3SS expression apart from AAA biosynthesis. It provides novel insight into the understanding of the biological function of AroQ for AAA biosynthesis via the shikimate pathway and its involvement in the T3SS expression in *R. solanacearum*.

## Data availability statement

The original contributions presented in the study are included in the article/Supplementary material, further inquiries can be directed to the corresponding author.

## Author contributions

TG and YW conceived and designed the experiments. QZ, BW, LH, and DY performed the experiments. DY, TL, and TG discussed results. YW wrote the manuscript. All authors contributed to the article and approved the submitted version.
