# Supplementary material for: Functional characterization of two 3-dehydroquinases of AroQ1 and AroQ2 in the shikimate pathway and expression of genes for the type III secretion system in Ralstonia solanacearum
Source: Front Microbiol. 2023 Apr 26;14:1186688. doi: 10.3389/fmicb.2023.1186688 (PMC10171560; doi:10.3389/fmicb.2023.1186688)
Supplement: Supplementary file 1 [file Table_1.docx]

Table S1. Primers used in this study

| primer | sequence | reference | |
| --- | --- | --- | --- |
| aroQ1A1B | ATGGATCCGCGTTCTTCGATGCCAG | | This study |
| aroQ1B1C | AGGGAAAAGTGGTGGCAGAGGTTTGACTGGATGGGCGAA | | This study |
| aroQ1A2C | TTCGCCCATCCAGTCAAACCTCTGCCACCACTTTTCCCT | | This study |
| aroQ1B2H | TCAAGCTTGATGATGAGATGCCGATGCC | | This study |
| aroQ2A1E | CTGAATTCGCAGTCGCTGACGCTGTTCG | | This study |
| aroQ2B1C | TGGCGCGCCGGGCGCCGGGAATTCAGTCTCCTGTCGGGCCG | | This study |
| aroQ2A2C | CGGCCCGACAGGAGACTGAATTCCCGGCGCCCGGCGCGCCA | | This study |
| aroQ2B2H | TCAAGCTTGAAGTAGTTGCGCAGCCA | | This study |
| glmsdown | GCGCTCAAGCTCAAGGAGATC | Zhang *et al*., 2011 | |
| Tn7R | CACAGCATAACTGGACTGATTTC | Zhang *et al*., 2011 | |
